# A BCI Gaze Sensing Method Using Low Jitter Code Modulated VEP

**DOI:** 10.3390/s19173797

**Published:** 2019-09-02

**Authors:** Ibrahim Kaya, Jorge Bohórquez, Özcan Özdamar

**Affiliations:** Neurosensory Engineering Laboratory, Department of Biomedical Engineering, University of Miami, Coral Gables, FL 33146, USA

**Keywords:** gaze sensing, SSVEP, BCI, c-VEP, transient VEP, QSS-VEP, deconvolution

## Abstract

Visual evoked potentials (VEPs) are used in clinical applications in ophthalmology, neurology, and extensively in brain–computer interface (BCI) research. Many BCI implementations utilize steady-state VEP (SSVEP) and/or code modulated VEP (c-VEP) as inputs, in tandem with sophisticated methods to improve information transfer rates (ITR). There is a gap in knowledge regarding the adaptation dynamics and physiological generation mechanisms of the VEP response, and the relation of these factors with BCI performance. A simple, dual pattern display setup was used to evoke VEPs and to test signatures elicited by non-isochronic, non-singular, low jitter stimuli at the rates of 10, 32, 50, and 70 reversals per second (rps). Non-isochronic, low-jitter stimulation elicits quasi-steady-state VEPs (QSS-VEPs) that are utilized for the simultaneous generation of transient VEP and QSS-VEP. QSS-VEP is a special case of c-VEPs, and it is assumed that it shares similar generators of the SSVEPs. Eight subjects were recorded, and the performance of the overall system was analyzed using receiver operating characteristic (ROC) curves, accuracy plots, and ITRs. In summary, QSS-VEPs performed better than transient VEPs (TR-VEP). It was found that in general, 32 rps stimulation had the highest ROC area, accuracy, and ITRs. Moreover, QSS-VEPs were found to lead to higher accuracy by template matching compared to SSVEPs at 32 rps. To investigate the reasons behind this, adaptation dynamics of transient VEPs and QSS-VEPs at all four rates were analyzed and speculated.

## 1. Introduction

Recently brain–computer interface (BCI) research has shown remarkable progress as witnessed by an increasing number of publications. The application areas of BCIs cover neural-rehabilitation or neuroprosthesis, locomotion, control, communication, entertainment, and gaming [1,2,3,4]. BCI provides a direct communication and control channel between the human brain and output devices to achieve a desired output function by using the control signals derived from the human brain [5]. Most of the BCI research is focused on increasing the information transfer rate (ITR), essentially increasing the throughput of the bridge between brain and device. Depending on the signal acquisition methods, Electroencephalography (EEG) based BCIs are gaining increased attention due to non-invasiveness and practical EEG headsets available on the market [6]. Research in the field of wearable sensors for EEG measurement systems for BCIs is critical for the expansion of BCIs. In [6], researchers introduced two multichannel EEG headsets in which all electrodes easily reach the scalp, and no preparation is required. In EEG based BCI applications, there are four main signal classes, namely slow cortical potentials (SCP), sensorimotor rhythms (SMR), P300 evoked potentials and visual evoked potentials (VEP) [1,2,3,5,7]. VEP based BCIs paradigms are common due to several advantages. These are mainly high ITR, simple setup, and little if any user training [8]. 

VEPs are generally named after stimulation types, such as flash VEP, pattern reversal VEP, pattern-onset-offset VEP [9]. In clinical testing, pattern-reversal VEP is the most preferred one due to having the least variability in the waveform and latencies [10]. VEP stimulus can be modulated by space, time, luminance, contrast, color, pattern, and depth [10]. VEPs can be grouped into two, depending on the stimulation rate. At low stimulation rates (<3–4 reversals per second (rps)) electrical activity on the occipital scalp elicits abrupt changes by the contrast reversing stimulus and then settles until the presentation of the next stimulus. This type of VEP is called ‘transient VEP’ (TR-VEPs) [11,12]. When the visual stimulus is presented at a higher rate, a periodic response containing frequency components with constant amplitude and phase, which is called ‘steady-state VEP’ or SSVEP [13,14], is generated. 

In VEP based BCIs, one question arises: independent or dependent? VEP is an exogenous signal generated by external visual stimulation, and it depends on the effector organs, such as the activity of the ocular muscles. For this reason, VEP BCIs are dependent BCIs. At the later stages of ALS disease, ocular muscle controls are lost and subjects loose gaze control. Thus, BCI must allow a mode of communication for these patients. A covert spatial attention mechanism was proposed for an independent VEP BCI [15]. Eye-tracking systems can be another option for the control signal. Eye trackers have been efficient for communication purpose so far. On the other hand, it is shown that EEG based (P300) 3 × 3 matrix BCI performed better than an eye-tracker and when it is combined with an eye-tracker, it achieved a higher performance than either [16]. Nezamfar et al. revealed that not only for the subjects with visual impairments but also for healthy individuals, VEP based gaze detection can be preferred instead of eye-tracking [17].

Depending on VEP modulation, three main VEP BCI types exist: time modulated VEP (t-VEP) BCI, frequency-modulated VEP (f-VEP) BCI, and pseudorandom code modulated VEP (c-VEP) BCI [18]. Both f-VEP and c-VEP BCIs produce high ITRs. Steady-state VEP (SSVEP) (f-VEP) based BCIs have the advantages of easy system configuration, excellent signal to noise ratio (SNR), immunity to interferences, minimal user training, easy quantification, and high ITR [5,18,19,20,21,22,23]. However, possible target frequencies are limited due to computer display refresh rate limitations, and the SSVEP has low amplitudes at high stimulation rates [5,19,23].

Pseudo random binary sequences (PRBS) based the c-VEP BCI paradigm offers promising results in terms of the ITR, accuracy, and use of many targets in applications [1,18,24]. Even though PRBS are mostly used in system identification, they are also used in physiological applications, such as deconvolution and BCI. One particular PRBS, the maximum length sequences or m-sequences were used by Sutter in a BCI application [25]. Although m-sequences have a-priori remarkable noise attenuation due to their sharp autocorrelation properties, they do not provide the expected SNR improvement in physiological recordings due to adaptation [26]. Working on BCI applications, Bin et al., found that when stimuli with sharp autocorrelation are used, the physiological system generates responses with lower autocorrelation sharpness, significantly reducing the a-priori effectivity of that BCI system [24]. Therefore, the knowledge of the adaptation characteristics of the visual system should be integrated into the design of the whole BCI system to achieve optimal performance. 

Low-jitter PRBSs have been successfully used to unveil the unitary responses of the visual system through pattern electroretinograms (PERG) and VEPs [27,28]. Low jitter stimuli elicit c-VEPs with a narrow spectrum, similar to the SSVEP and are named as Quasi SSVEP (QSS-VEP); they have unique features, such as the capability for deconvolution of transient VEPs (TR-VEPs), immunity to adaptation, and shared brain generators of the SSVEP. 

The use of a physiologically adapted low jitter PRBS stimuli in a BCI gaze sensor may capture advantages of both f-VEPs and c-VEPs paradigms, reducing their shortcomings. For this reason, in this study, we developed a dual-display BCI gaze sensor based on specially designed low-jitter PRBS codes eliciting QSS-VEP and adopted the template matching as the detection method.

## 2. Materials and Methods

A two target right and left gaze sensing systems was developed using two independent visual fields (right field RF and left field LF) on a fast Visual Display Unit (VDU). Four stimulation rates (10, 32, 50, and 70 rps) were explored in a normal subject population. For each rate, the detection was evaluated using QSS-VEP and TR-VEPs. For the rates of 10 and 32 rps, the SSVEPs were also evaluated. Fifty and 70 rps SSVEPs were excluded due to low amplitudes [29]. Using raw EEG recordings, the system performance, in all conditions, was quantified off-line by using receiver operating characteristic (ROC) curves, areas under the curves (AUC), accuracies, and ITRs.

### 2.1. Subjects

A total of 8 subjects (5M, 3F) with no history of neurological impairment participated in this study. The subject ages ranged from 18 to 31. All the subjects had either normal or corrected-to-normal vision and were right eye dominant. They signed informed consent forms approved by the University of Miami Institutional Review Board (IRB) before the participation. 

### 2.2. Stimuli Design and Stimulator Display

**Figure 1:**
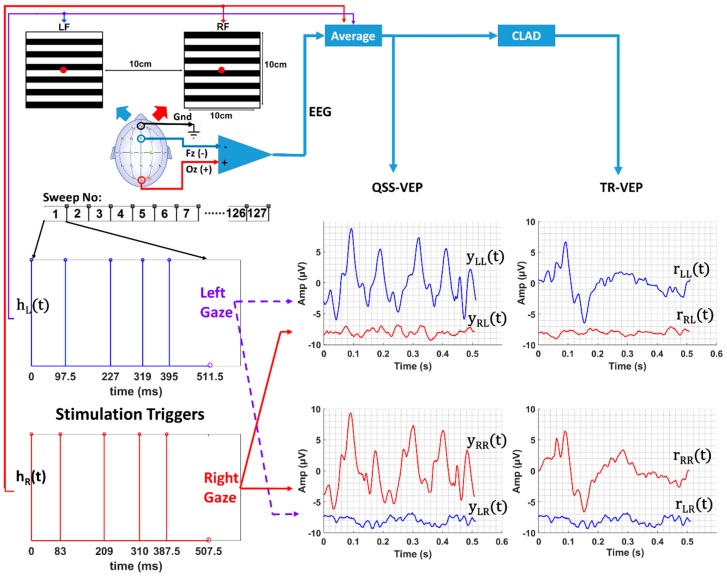
Overall system setup and the experimental procedure are shown for a 10 rps stimulation. The stimulator is shown at the top left, driving sequences for the left field (LF) and right field (RF) are shown at the bottom left, the quasi-steady-state (QSS) and transient responses from subject 5 are shown in the bottom right figures. Interference signals after averaging 127/128 sweeps in one file for left/right, $y_{RL}\left(t\right)$ and $y_{LR}\left(t\right)$, are also displayed to highlight the orthogonality of the sequences. Refer to Section 2.4 and abbreviation list at the end of the paper for the abbreviations used in the figure.

The display consisted of two fast switchings on-demand LED-based regions for left (Left Field, LF) and right targets (Right Field, RF) (Figure 1). Subjects were placed 1 meter away from the stimulator display to ensure both targets lie in the foveal field of view. The stimulator provided two target stimuli, each covering a visual field of 5.7° × 5.7°. The luminance of the display was 580 Cd/m^2^. The stimulus pattern of the targets consisted of 12 horizontal black and white bars with a spatial frequency of 1.03 cycles/degree. The display was built with the same LED display technology that was described in [27] and [30]. The ON/OFF rising and falling times were less than 105 µs, and it was used in their experiment to obtain 78 rps responses [27]. Odd and even bars of the display were driven by the controllers of the digital output simultaneously to make the pattern reversal synchronous. Right and left fields were driven by two stimuli of slightly different length and jitter but at a similar rate (Table 1). The sequences were designed to be orthogonal to obtain individual responses to each display field. Low jittered, low noise amplification sequences similar to [27,28,31] were used. These sequences have low jitter and thus, resemble the steady-state iso-chronic stimulation sequences. Moreover, they are non-singular in the frequency domain and allow deconvolution to extract transient VEPs (See [32] for deconvolution). A cue light blue for Left, red for Right, green for Center was lit for an indication of gaze direction to the subjects. 

### 2.3. Experimental Setup and EEG Recording

Although many electrodes can be used to obtain better accuracies by spatial filtering techniques, we aimed to keep it simple to see only the effects rate and stimuli dependent physiological phenomena. In [23], they found that all the subjects had the maximum signal at the Oz electrode location. From the previous recordings we have done so far in this laboratory, we obtained good results with the Oz–Fz configuration, and we continued on this bipolar derivation. 

Three passive Ag/AgCl electrodes were used in the experiments for three electrode locations (Oz (+), Fz (-), and forehead (gnd)). Electrode impedances were checked and kept below 7 kΩ. EEG was recorded with an Intelligent Hearing System (IHS, Miami, FL, USA) data acquisition module (16 bits) at a sampling rate of 2 kHz. The analog signal was bandpass filtered with 1 to 300 Hz (6 dB/octave) filters. Raw EEG data along with stimulation triggers and synchronization cues, were stored for off-line analysis.

In this study, we collected between 4 and 6 trials for all the conditions. Some of the trials were used for template generation and the remaining for testing. We aimed to achieve a similar sweep number for the templates. Within the QSS comparison, we used 124 to 128 sweeps in template generation. For 10 rps SS/QSS comparison, we selected 117 to 127 sweeps; for 32 rps QSS/SS comparison, 93 to 96 sweeps were used. The acquisition order was 10 rps, 32 rps, 50 rps, and 70 rps, consecutively. Template acquisition was done by averaging EEG sweeps, for number of sweeps, refer to Table 1. During the recordings, the operator monitored the experiment to ensure signal quality, no powerline interference, subject’s cooperation, subject’s gaze. In the event of poor cooperation by subject, sleepiness or excessive eye blinks, the recording was halted and either repeated or postponed for another time. Between recordings, subjects had 30 s rest intervals.

### 2.4. EEG Offline Processing

Template VEPs were computed by conventional ensemble averaging of the epochs (sweeps) of the same condition; artifacts and noisy sweeps with peak to peak amplitudes >80 µV were rejected. The residual noise was estimated using the +/- averaging method as the average of the odd sweeps minus the even sweeps [33,34]. SNR was computed as the ratio of the signal power to noise power in dB.

For one cycle/trial of stimulation when the subject gazed at one stimulation region, the response to the other stimulation region is almost zero due to orthogonality which can be seen from $y_{RL}\left(t\right)$(red) and $y_{LR}\left(t\right)$(blue) plots in Figure 1.

Left and right display regions were driven by $h_{L}\left(t\right)$ and $h_{R}\left(t\right)$ stimulation sequences, respectively, as shown in Figure 1. The 10 rps QSS-VEPs for left and right regions were obtained by averaging the EEG data with window lengths of 1024 and 1016 points or 512 ms and 508 ms windows. For the other reversal rates, the window sizes or sweep durations are given in Table 1. The QSS-VEP responses are hypothesized to result from the convolution of the stimulation sequences $h_{L}\left(t\right)$ or $h_{R}\left(t\right)$ and ideal transient response at that particular adaptation rate. This can be seen from Equations (1) and (2).

(1) $$y_{LL}\left(t\right)=h_{L}\left(t\right)*r\left(t\right)\Rightarrow Y_{LL}\left(f\right)=H_{L}\left(f\right)*R\left(f\right),$$

(2) $$y_{RR}\left(t\right)=h_{R}\left(t\right)*r\left(t\right)\Rightarrow Y_{RR}\left(f\right)=H_{R}\left(f\right)*R\left(f\right).$$

Using frequency domain deconvolution [26], the transient responses for the right field ($r_{RR}\left(t\right)$) and left field stimulation and left gazes ($r_{LL}\left(t\right)$) were obtained, as shown in Equations (3) and (4).

(3) $$R_{RR}\left(f\right)=\frac{Y_{RR}\left(f\right)}{H_{R}\left(f\right)}\Rightarrow r_{RR}\left(t\right)=IFFT\left(\frac{Y_{RR}\left(f\right)}{H_{R}\left(f\right)}\right),$$

(4) $$R_{LL}\left(f\right)=\frac{Y_{LL}\left(f\right)}{H_{L}\left(f\right)}\Rightarrow r_{LL}\left(t\right)=IFFT\left(\frac{Y_{LL}\left(f\right)}{H_{L}\left(f\right)}\right).$$

The stimulation sequences were designed to have similar jitter characteristics; this led to very similar transient responses of left and right fields.

By averaging the multiple EEG data segments of left or right sequence lengths, it was possible to extract QSS-VEP responses to right and left targets (see Figure 1) for different data sizes. Since stimulation sequences were nonsingular, the deconvolution method in the frequency domain was applied to obtain TR-VEP responses. QSS-VEP and TR-VEP templates were computed by averaging the two EEG files for 10 rps and 50 rps, similarly three EEG files for 32 rps and 70 rps for each gaze direction. 

### 2.5. Gaze Detection and ROC Estimation

The gaze detection process involved cross-correlation between the reference templates and the signal. We utilized Pearson’s product-moment correlation coefficient between the subject’s template VEP and the subject’s actual test data. The correlation coefficient was compared to a threshold for decision; if the correlation coefficient was higher than the threshold, the signal matched the template’s gaze direction. For this purpose, templates for each gaze and rate condition were extracted following the training sessions. Proper calculation and selection of threshold values from cross-correlation coefficients were critical in the gaze detection. The ROC is a curve representing the performance of the classifier; by the change of operation threshold value, the ROC determines the probability of error and accuracy (true positive ratio) in the system. After the files were recorded and the templates were ready to be used in the comparison, ROC curves were calculated to evaluate the performance of the classifier. To compute ROC curves, two classes of files (left and right gaze files) were fed into an algorithm where left or right templates were used as references. Since file gaze conditions were known, true positives, false positives, true negatives, false negatives were available while the threshold was varied from −1 to 1 by steps of 0.05 for the correlation coefficient. ROC curves were computed for 0.5 s, 1 s, 1.5 s, 2 s, 2.5 s, 3 s, 3.5 s, 4 s of data length.

## 3. Results

Overall system performance was evaluated by offline analysis. This covered ROC curves and areas, accuracies, ITR computations, plots, and comparisons. Generally, *t*-test paired two samples for means were used to examine the effects of various conditions on the performance, particularly on accuracy and ITR. The effect of gazing left or right stimulus was tested by computing accuracies. It was found that statistically there was no significant difference in the mean accuracies by left or right gaze using single sweep (0.5 s) QSS-VEP signature at all rates. Therefore, for the same conditions left and right gazed performances were averaged to generate a single parameter to represent the performance at that particular condition. This reduced the number of comparisons and hence, complexity in the analysis.

There were many signatures to test the system performance with. For 10 rps stimulation, these were mainly the combinations of right and left, QSS-VEP, and TR-VEP responses. It followed the same signatures for 32 rps, 50 rps, and 70 rps stimulation. By averaging left and right QSS-VEP performances in terms of accuracy or ROC area, an average accuracy or ROC area was computed for each rate of QSS (10, 32, 50, 70 rps) and steady-state (SS) conditions (10 and 32 rps).

### 3.1. VEP Morphology by Rate Adaptation

**Figure 2:**
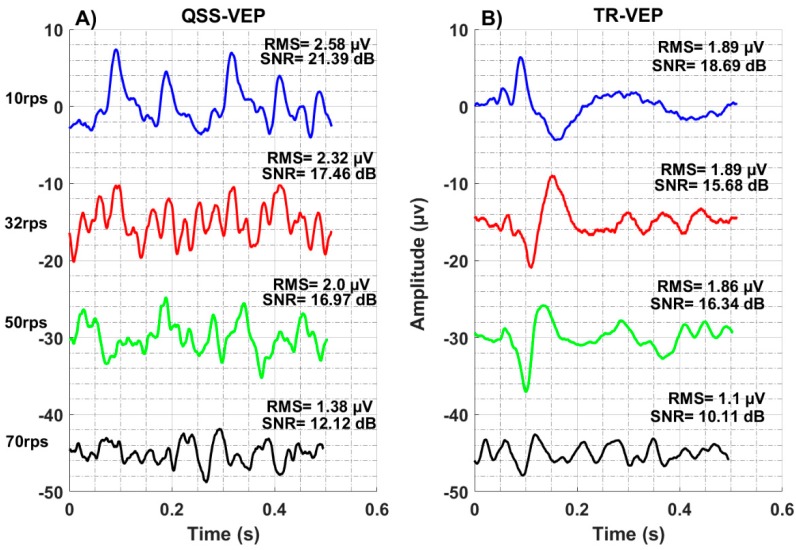
Population average left field quasi-steady-state-visual evoked potentials (QSS-VEP) (**A**), and transient-visual evoked potentials (TR-VEP) (**B**) plots are shown. (A) 10 rps, 32 rps, 50 rps, and 70 rps QSS-VEPs are at the left. (B) TR-VEPs are at the right. Note the morphology change in the TR-VEPs by rate from 10 rps to 32 rps.

The recording electrode sites might reveal different signals depending on the adaptation characteristics of dominant neural populations over particular sites [35]. Four rates of 10 rps, 32 rps, 50 rps, and 70 rps QSS-VEPs are shown in Figure 2A. It can be observed that 70 rps QSS-VEP has smaller peaks compared to others. In Figure 2B, transient TR-VEP waveforms are shown. While 10 rps TR-VEP had characteristic early peaks and negativities, 32 rps, 50 rps, and 70 rps had totally different waveforms. There a positive peak replaced the negativity around 120 to 150 ms. When we compare the TR-VEP amplitudes, 32 rps had the largest amplitude 12 µV, 50 rps had 10.9 µV, 10 rps had 10.5 µV, and 70 rps had the minimum 5.2 µV amplitude.

### 3.2. Offline Analysis and Performance

Template matching was adopted as the method for target recognition. Threshold values for right and left target detection were pulled from ROC curves as the threshold index value corresponding to max ROC AUC. Once thresholds were set and template signals were uploaded, files with known status were tested with the system, and the results were plotted.

There are many metrics to evaluate the performance of a BCI system. Thompson et al., compared these metrics of accuracy or error rate, Cohen’s Kappa coefficient, confusion matrix, mutual information, and ITR in terms of throughput, categorical output, unbiased/biased, practicality [36]. They suggested ITR as the optimum metric for level-1 BCI system. BCIs consisting of only the control module, difficult to work at high speed, accuracy, and ease of use, which remains mainly used in research, medical, and clinical context, are referred to as level-1. Our system was tested offline with QSS-VEP and TR-VEP signatures and for all stimulation rates. ITR and accuracies were used in this evaluation. ITR can be calculated by the following Equation (5):

(5) $$ITR=\left[log_{2}\left(N\right)+P\;log_{2}\left(P\right)+\left(1-P\right)\;log_{2}\left(\frac{1-P}{N-1}\right)\right]\frac{60}{T}.$$

*P* is the probability of correct selection, *N* is the number of choices, and *T* (seconds/selection) time required to select a choice/target [36]. Its unit is given in bits per minute (bpm). The ITR simply follows the accuracy of the classification, the higher the accuracy, the bigger is the ITR. There is a limit of the ITR by selection time and maximum accuracy of 1. For a two target system, at maximum accuracy and for 0.5 s selection time, max ITR is120 bpm. 

In the experiments subject 6 achieved a remarkable accuracy of 0.98 and a corresponding ITR of 100 bpm at 32 rps 0.5 s data and QSS-VEP right gazing BCI switch condition. QSS-VEP and TR-VEP ROC curves for 50 rps and left gaze conditions were compared. For data durations 0.5 s and 1.5 s, 50 rps mean ROC areas were found as 0.86 and 0.92, respectively, for the left QSS signal. Similarly, for left TR-VEP, 0.5 s and 1.5 s data durations, accuracies were 0.73 and 0.80, respectively. It should be noted that the ROC area increases by the increase in data size. Inclusion of extra sweeps in the average increases the SNR and better ROC area and accuracy are achieved.

**Figure 3:**
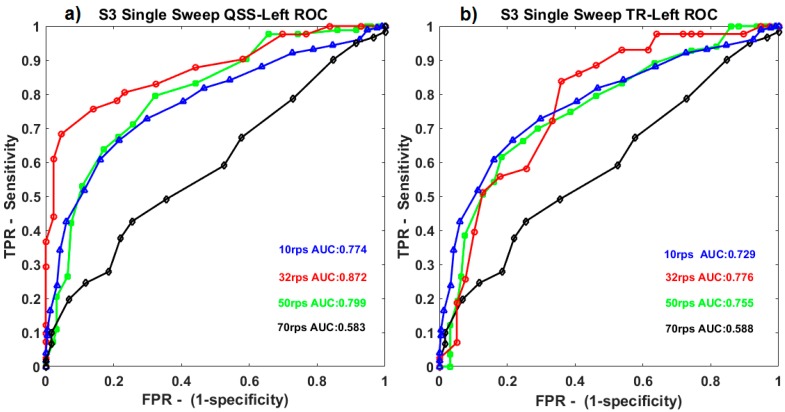
Subject 3 (S3) single sweep 0.5-second condition. (**a**) Left QSS-VEP receiver operating characteristic (ROC) curves and areas for all the rates are shown at the left. (**b**) Left TR-VEP ROC curves and areas for all the four rates are shown at the right.

When ROC curves for QSS-VEP and TR-VEPs were compared, for single sweep (0.5 s), QSS-VEPs were found to perform better than TR-VEPs for 32 rps stimulation (*p* < 0.05) (See Figure 3). Classifier performances for TR-VEPs using 0.5 to1 s sweep data were compared in Figure 3b. It can be observed from Figure 3 that among S3 ROC curves for different rates, 32 rps had the largest ROC area for both left QSS-VEP and left TR-VEP. Ten reversals per second and 50 rps had similar ROC areas for QSS-VEP, 70 rps had the minimum ROC area for both QSS-VEP and TR-VEP which summarizes the system performance at these rates. The QSS-VEP paradigm boosts the ROC area performances obtained by TR-VEPs by 12.4% for 32 rps for S3. 

Table 2 presents the single sweep (0.5 s) QSS-VEP accuracy, ITR, mean testing signal SNR, and root mean square (RMS) values. Single sweep SNRs were computed using the subject individual VEP references for each condition as the signal estimate, and the noise was computed as the difference between single sweep and the reference. These noise and signal energies were averaged to get the mean SNR for 0.5 s data. It can be observed from Table 2 that when the SNR was high within the subject values, it corresponded to high accuracy and ITR. On the other hand, if one subject had higher SNR than the other one, accuracy and ITR were higher as well. The 32 rps QSS-VEP had larger average single sweep SNR (*p* < 0.05) and thus, higher accuracy and ITR. The single sweep average SSVEP SNRs were calculated similarly and found to be 0.31 dB for 10 rps, and 0.01 dB for 32 rps which were much smaller compared to QSS-VEP counterparts.

**Figure 4:**
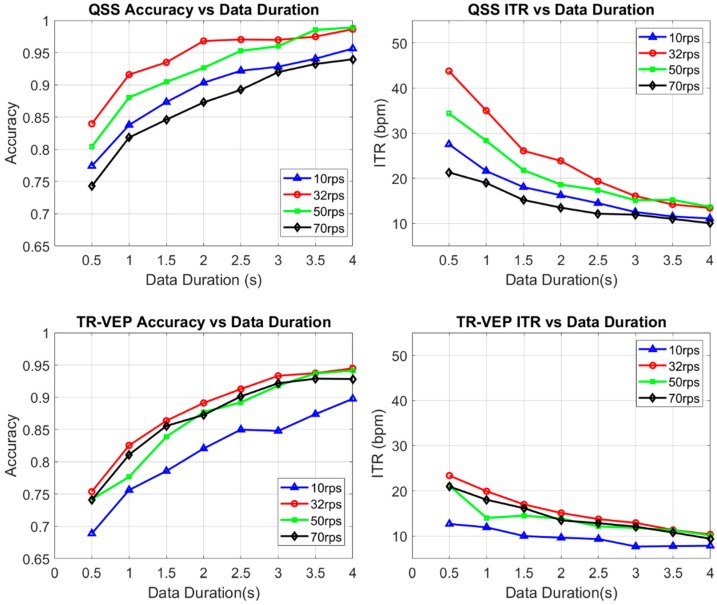
QSS-VEP performances for left and right gazes were averaged as single QSS-VEP performance parameter at each rate. For single sweep 0.5 s, 1 s, and 1.5 s, 32 rps had the highest accuracy and ITR performance (*p* < 0.05) with QSS-VEP signature while 70 rps had the lowest performance (*p* < 0.05). For the eight subject population, average ITR values for QSS-VEP (top-right) and TR-VEPs (bottom-right) were plotted for each rate and data durations.

As mentioned previously, the left and right QSS-VEP performances were combined to create a single parameter for that condition, which revealed the rate-dependent characteristics easily in one figure. See Figure 4 for the rate effect on QSS-VEP and TR-VEP signals. It was found that for QSS-VEP 32 rps had the biggest accuracy and ITR among all rates for 0.5 s data. QSS-VEP ITRs were bigger than TR-VEP ITRs. On the other hand, TR-VEP ITR performances can be seen from the bottom right panel in Figure 4 which shows that, except for 10 rps, the remaining rates had ITR values around 20 to 23 bpm for 0.5 s data duration. Ten reversals per second had low TR-VEP accuracy performance and low ITR around 14 bpm with single sweep data. The lower accuracy of 10 rps can be attributed to the alpha band activity interference, which was reported in [29] and [37].

**Figure 5:**
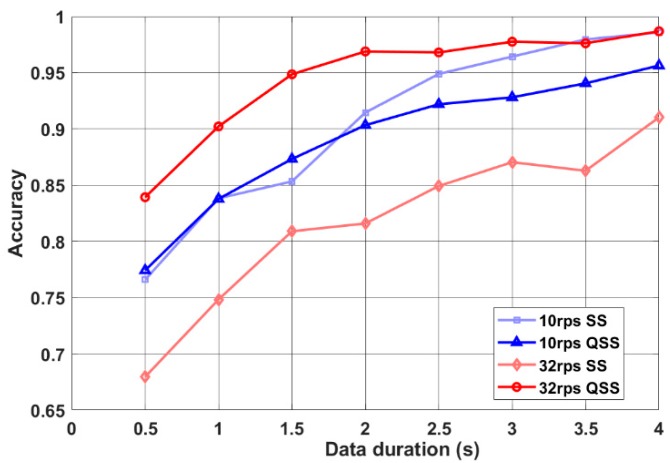
QSS-VEP and steady-state visual evoked potentials (SSVEP) performances are compared. QSS-VEPs performed better than SSVEPs for short data durations.

When 10 rps QSS and steady-state (SS) performances were compared, QSS-VEP 10 rps had a similar performance with 10 rps SS performance as seen in Figure 5. On the other hand, when 32 rps QSS was compared to SS, SS performed much lower than 32 rps QSS (*p* < 0.05) for all data durations. QSS-VEP 32 rps paradigm had on the average 23.5% higher accuracy than the SSVEP paradigm for 0.5 s data. This shows the improvement in high rate SSVEP accuracies by QSS paradigm.

## 4. Discussion

Although SSVEP based BCIs demonstrated their excellence in ITRs, performances of pattern-reversal c-VEP and transient-VEP based BCIs can be improved. Since there is a limited number of studies with pattern-reversal VEP, we proposed a research into different reversal rates and the template matching performance of the pattern reversal c-VEP or particularly low jittered QSS-VEPs. For TR-VEP ROC comparisons, 10 rps performed low compared to other rates. This might be attributed to the interference with the alpha band at 10 rps. There existed a large intra-subject variability in the accuracies and ITRs. It was found that user variation could be reduced by selecting subject-specific electrode location, stimulus frequency, and amount of EEG data used in the signature computation [8]. On the other hand, in addition to Oz, TP9 and TP10 electrode areas behind the ears also gave strong responses to the color and luminance stimuli [38]. The advantage of this method is that it provides more comfortable electrode montages due to no hair on these areas. It is very important for BCI systems to be mounted on the subject easily. 

Another source of variation could be attention and cooperation during the particular tasks; if the user lacked attention during target gazing, the resultant target VEP characteristics would be affected. Hence, accuracy and ITRs would be influenced adversely. 

For the fatigue problem, on the other hand, it is known that SSVEPs cause fatigue and epileptic seizures [39]. However, with high rate SSVEP stimulation (above 30 Hz), fatigue and seizures can be reduced [40]. In addition to the high rate, Xie et al., demonstrated that visual fatigue can be decreased by using random visual noise around the visual targets [41].

However, on the overall performance, 32 rps QSS-VEP paradigm led to the highest accuracy values and ITRs in the offline analysis compared to 10 rps, 50 rps, and 70 rps. One of the reasons behind this is that the number of visible peaks with 32 rps stimulation in the resultant QSS-VEP was higher than the number of visible peaks with other QSS stimulation rates. However at 50 rps and 70 rps, due to convolution and superposition of transient VEPs, the resulting QSS-VEPs did not elicit that many peaks contributing to template matching. Another aspect of the research was a comparison of SSVEP to QSS-VEP in template matching accuracy. As mentioned previously, SSVEPs had smaller peaks at high rates [29]. QSS-VEPs, on the other hand, had multiple peaks and higher amplitudes compared to SSVEPs. 

QSS stimulation improved the SNR performance due to jitter in the paradigm, and this jitter canceled out adverse effects of adaptation on signal peaks. QSS-VEP generated larger peaks at higher rates where steady-state responses were diminished by adaptation. The low jitter design of QSS stimulation allowed the extraction of base/unitary TR-VEPs responses. If TR-VEPs had bigger amplitudes at particular rates, it was due to the boosting effect of special QSS stimulation. As compared QSS-VEPs at 32 rps performed significantly (*p* < 0.05) better than 32 rps SS counterparts for 1, 2, or 3 sweeps averages. 

The performance of the gaze sensors is not only affected by the SNR but also by the saliency and the number of the peaks in the templates. Among all the investigated rates and stimulation characteristics, QSS-VEP at 32 rps performed better. This paradigm provides a good compromise between SNR and richness of the template. The rich 32 rps signature (Figure 2) was generated by the sharp characteristics of the transient response convolved with the stimulation sequence. To optimize the gaze sensor performance, both the underlying physiological knowledge and the sequence characteristics should be used as guidelines in the design.

## 5. Conclusions

This work aimed to present a better design of stimulation sequences and use of the QSS stimulation paradigm to demonstrate the importance of designing the stimulations sequences in line with the transient VEPs obtained at the rate of stimulation. Offline analysis was given for this purpose, and arguments were supported by these results. However, for the final evaluation of the proposed method, online accuracy, and ITR analysis can be carried out. It is projected to work on online testing of the system in future works.

This research revealed that QSS-VEP paradigm offers a feasible alternative to the methods applied to c-VEP or SSVEP based BCIs. The advantages of the QSS-VEP method are that it increases the accuracy of the SSVEP template matching at the same reversal rates, and it enables extraction of clinically significant TR-VEP from low jittered QSS-VEPs. By special design of low jitter QSS sequences, it was possible to enhance accuracy at higher rates compared to rates interfering with the alpha band. Thirty-two reversals per second achieved the maximum boosting effect by the QSS-VEP, which led to higher accuracy performances in template matching based dual-target BCI switch. The results can also be improved by using more electrodes and spatial filtering methods. However, the main issue addressed in this study is to use a simple technique to investigate the underlying physiologic phenomena and its relation to BCI performance. Another aspect to be considered is visual fatigue; the fatigue resulting from the QSS paradigm can be compared with the SSVEP fatigue at the same stimulation rate in future studies.

## Figures and Tables

**Table 1:** Stimulation properties for quasi-steady-state (QSS) and steady-state (SS) stimulation paradigms and sweep number used for template generation.

Signal	Nominal Rate (rps)	Mean Rate (rps)	Field Left-LF Right-RF	Sweep Duration (ms)	Trial Sweep Count	Template Acquisition Sweep Number
QSS	10	9.76	LF	512	127	127
		9.84	RF	508	128	128
QSS	32	31.25	LF	512	31	124
		30.25	RF	496	32	128
QSS	50	49.6	LF	504	62	124
		52.4	RF	496	63	126
QSS	70	70.5	LF	496	32	128
		70.3	RF	512	31	124
QSS	10	9.76	LF	512	127	127
		9.84	RF	508	128	128
SS	10	10	LF	500	39	117
		10.5	RF	487.5	40	120
QSS	32	31.25	LF	512	31	93
		30.25	RF	496	32	96
SS	32	31	LF	512	31	93
		32	RF	496	32	96

**Table 2:** Single sweep (0.5 s) quasi-steady-state-visual evoked potentials (QSS-VEP) accuracy, information transfer rates (ITR), mean signal to noise ratio (SNR), and root mean square (RMS) overall average values for all rates and eight subjects (S1–S8).

**Subject**	**Accuracy**							
	**10 rps**		**32 rps**		**50 rps**		**70 rps**	
	**L**	**R**	**L**	**R**	**L**	**R**	**L**	**R**
S1	0.82	0.77	0.84	0.79	0.89	0.78	0.89	0.73
S2	0.90	0.92	0.92	0.93	0.85	0.83	0.89	0.88
S3	0.73	0.71	0.82	0.78	0.73	0.66	0.58	0.67
S4	0.83	0.78	0.84	0.79	0.78	0.84	0.70	0.74
S5	0.75	0.71	0.79	0.87	0.76	0.79	0.71	0.67
S6	0.88	0.94	0.95	0.98	0.82	0.87	0.72	0.86
S7	0.70	0.61	0.72	0.72	0.65	0.71	0.61	0.60
S8	0.62	0.71	0.83	0.86	0.84	0.86	0.84	0.79
Mean (STD)	0.77 (0.10)		0.84 (0.07)		0.79 (0.07)		0.74 (0.10)	
**Subject**	**ITR (bpm)**							
	**10 rps**		**32 rps**		**50 rps**		**70 rps**	
	**L**	**R**	**L**	**R**	**L**	**R**	**L**	**R**
S1	38.39	27.27	44.17	30.11	58.58	28.78	60.37	18.68
S2	64.10	72.59	70.48	75.64	46.52	41.08	61.10	55.45
S3	18.85	15.91	38.65	29.22	19.54	8.91	2.40	10.09
S4	41.35	29.66	44.75	31.95	29.44	43.31	14.54	20.79
S5	22.08	16.38	31.02	54.44	24.60	31.25	15.75	9.84
S6	57.52	79.76	85.12	99.76	37.61	53.44	17.84	48.65
S7	14.10	4.30	17.67	17.35	8.02	15.45	3.92	3.35
S8	5.03	14.99	41.63	49.58	42.74	48.95	42.46	31.48
Mean	26.64		43.88		31.02		20.79	
**Subject**	**SNR (dB)**							
	**10 rps**		**32 rps**		**50 rps**		**70 rps**	
	**L**	**R**	**L**	**R**	**L**	**R**	**L**	**R**
S1	0.77	0.67	0.42	0.08	0.92	0.74	0.58	0.10
S2	1.08	0.97	1.56	1.42	0.66	0.77	0.95	0.70
S3	0.29	0.11	0.19	0.16	0.23	0.06	−0.11	0.07
S4	0.57	0.44	0.66	0.35	0.45	0.23	0.12	0.15
S5	0.24	0.22	0.35	0.29	0.30	0.24	0.13	−0.02
S6	0.55	0.64	0.82	1.05	0.24	0.31	0.09	0.28
S7	0.13	−0.16	0.09	0.10	0.04	−0.13	−0.04	−0.13
S8	−0.01	−0.24	0.28	0.43	0.31	0.76	0.23	0.16
Mean	0.39		0.52		0.38		0.20	
**Subject**	**Average QSS-VEP RMS (µV)**							
	**10 rps**		**32 rps**		**50 rps**		**70 rps**	
	**L**	**R**	**L**	**R**	**L**	**R**	**L**	**R**
S1	4.98	4.48	4.10	3.26	4.75	4.38	4.30	2.89
S2	3.85	3.82	4.45	4.60	2.86	2.84	3.36	3.38
S3	3.25	3.61	4.07	3.44	3.34	3.05	1.95	2.85
S4	2.26	2.12	2.28	1.95	2.17	2.20	1.20	1.38
S5	2.51	2.56	2.71	3.02	2.22	2.63	2.09	1.61
S6	4.67	4.90	4.80	5.15	3.19	3.17	2.37	2.77
S7	2.22	2.20	2.33	2.23	1.77	1.65	1.53	1.41
S8	1.81	1.91	2.68	3.04	3.08	3.25	2.02	2.10

## Abbreviations

ALS	Amyotrophic Lateral Sclerosis	QSS-VEP	Quasi-Steady-State VEP
AUC	Area Under the Curve	RF	Right Field
BCI	Brain Computer Interface	RL	Right seq. averaging of Left gaze
bpm	Bits per minute	RMS	Root mean square
c-VEP	Code modulated VEP	ROC	Receiver Operating Characteristics
EEG	Electroencephalography	rps	reversal per second
FPR	False Positive Rate	RR	Right seq. averaging of Right gaze
f-VEP	Frequency-modulated VEP	r(t)↔R(f)	Unitary transient response
h(t)↔H(f)	Stimulus sequence	SCP	Slow Cortical Potential
IFFT	Inverse Fast Fourier Transform	SMR	Sensorimotor Rhythm
ITR	Information Transfer Rate	SNR	Signal to Noise Ratio
LF	Left Field	SSVEP	Steady-state VEP
LL	Left seq. averaging of Left gaze	TR-VEP	Transient Visual Evoked Potential
LR	Left seq. averaging of Right gaze	TPR	True Positive Rate
m-sequence	Maximum length sequence	t-VEP	Time modulated VEP
PERG	Pattern Electroretinogram	VEP	Visual Evoked Potential
PRBS	Pseudo Random Binary Sequence	y(t)↔Y(f)	VEP response
